# The predictive value of admission blood glucose to serum albumin ratio for futile recanalization after successful interventional recanalization in acute cerebral infarction with large vessel occlusion

**DOI:** 10.3389/fneur.2024.1442979

**Published:** 2024-12-30

**Authors:** Wensheng Zhang, Jie Li, Hongxing Zhou, Weifang Xing, Kaifeng Li, Yudi Li, Jinzhao He, Xiongjun He, Yajie Liu, Li Ling

**Affiliations:** ^1^Department of Neurology, Heyuan People’s Hospital, Guangdong Provincial People's Hospital Heyuan Hospital, Heyuan, Guangdong, China; ^2^Department of Neurology, Shenzhen Hospital, Southern Medical University, Shenzhen, China; ^3^The Third School of Clinical Medicine, Southern Medical University, Guangzhou, China; ^4^Heyuan Key Laboratory of Molecular Diagnosis & Disease Prevention and Treatment, Doctors Station of Guangdong Province, Heyuan People's Hospital, Heyuan, Guangdong, China

**Keywords:** admission blood glucose to serum albumin ratio, large vessel occlusion, acute cerebral infarction, interventional recanalization, futile recanalization

## Abstract

**Aims:**

We aim to explore the predictive value of admission blood glucose to serum albumin ratio (AAR) for futile recanalization after successful interventional recanalization of acute cerebral infarction.

**Methods:**

We retrospectively collected the data of patients suffered from acute cerebral infarction due to anterior circulation large vessel occlusion and received successful interventional recanalization from January 2019 to November 2023. Statistical analysis of clinical data was conducted using SPSS 26.0.

**Results:**

A total of 452 patients were included in the study. There were statistically significant differences in mRS score and futile recanalization rates among the three groups of patients at 3 months after surgery according to AAR tertile. In the multiple logistic regression analysis, there were statistically significant differences in Diabetes, grade of NIHSS score at admission, AAR tertiles, good collateral circulation and Pathogenesis. The Receiver Operating Characteristic curve (ROC) curve of AAR predicting futile recanalization was drawn with an AUC of 0.582 and a 95% confidence interval of 0.529–0.634. After combining grade of NIHSS score at admission, AAR tertiles and good collateral circulation, a ROC curve was drawn to predict futile recanalization, with an AUC of 0.907 and a 95% confidence interval of 0.879–0.936.

**Conclusion:**

AAR is a new composite indicator for predicting futile recanalization in patients with acute cerebral infarction with anterior circulation large vessel occlusion. The combination of grade of NIHSS score at admission, AAR tertiles and good collateral circulation has a high predictive power for futile recanalization.

## 1 Introduction

Acute cerebral infarction with large vessel occlusion has a high incidence rate, mortality rate and disability rate (1). Interventional recanalization is one of the most important treatment methods for acute cerebral infarction with large vessel occlusion. Although the success rate of recanalizating the occluded blood vessels can reach up to 90%, the proportion of futile recanalization after 3 months of surgery can be as high as 50–60% (2–9). It was defined as futile recanalization when patients cannot achieve independent neurological function with a mRS score of 3–6 points at 3 months after surgery (10). The existence of futile recanalization seriously weakens the efficacy of interventional recanalization. It is very necessary to establish a simple and accurate prediction model for futile recanalization, so as to early predict high-risk populations for futile recanalization and do early intervention to improve patients’ prognosis. Previous studies had shown that elevated blood glucose level at admission was associated with futile recanalization in patients (11, 12). Some studies had also found that lower serum albumin was associated with adverse outcomes at 3 months (13, 14). In addition to being a nutritional indicator, serum albumin is also a multifunctional protein related to diabetes, inflammation and thrombosis (15–17). Admission blood glucose reflects the need for blood glucose control, and serum albumin levels can guide the treatment of malnutrition, inflammation and thrombosis. The correlation between the Admission blood glucose to serum albumin ratio (AAR) and futile recanalization after successful interventional recanalization in acute cerebral infarction with large vessel occlusion is still unclear. The aim of this study is to evaluate the predictive value of AAR for futile recanalization after successful interventional recanalization in acute cerebral infarction with anterior circulation large blood vessel occlusion.

## 2 Materials and methods

### 2.1 Study population

**Figure 1:**
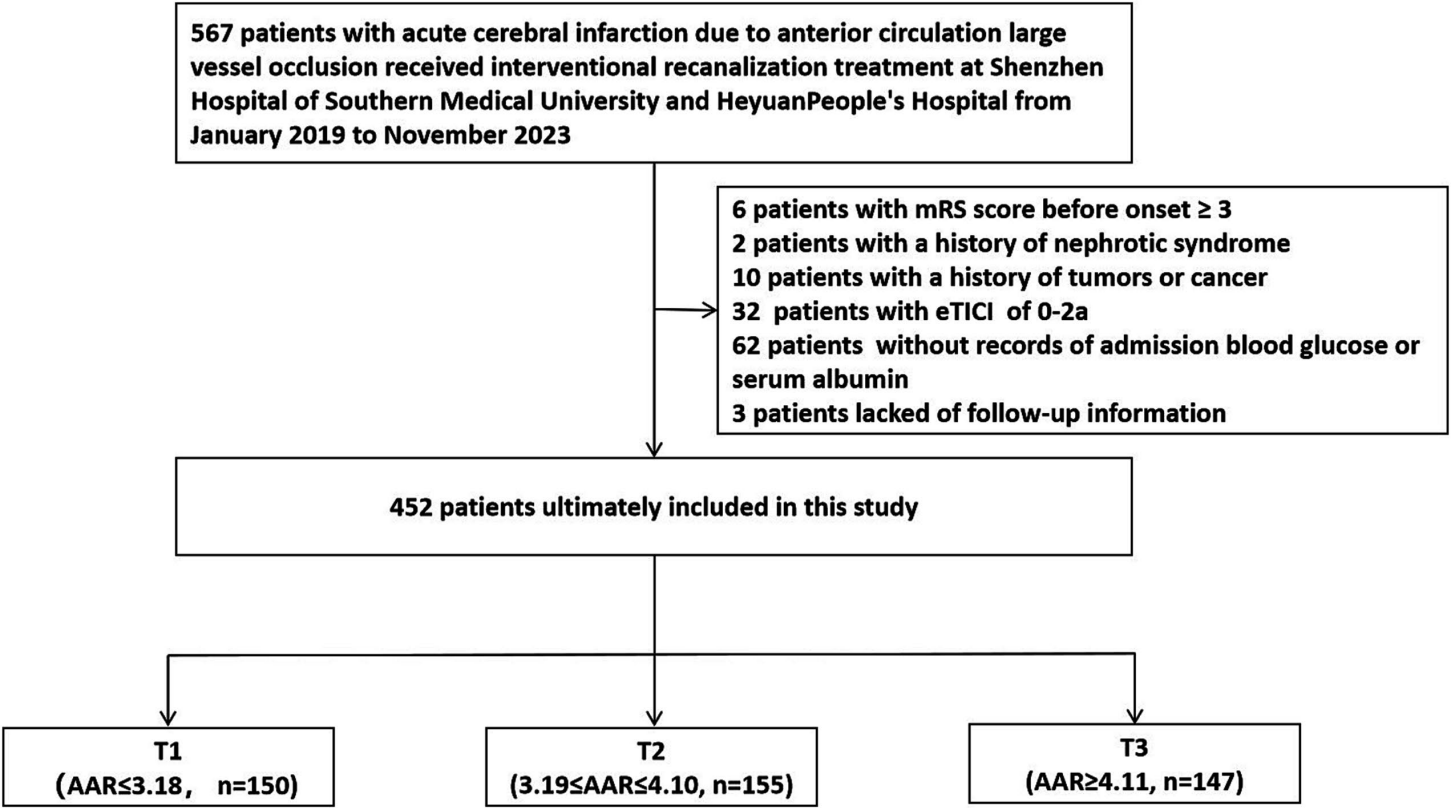
Research flowchart. eTICI, extended thrombolysis in cerebral infarction; AAR, Admission blood glucose to serum albumin ratio.

We retrospectively analyzed 452 patients with acute cerebral infarction caused by anterior circulation large vessel occlusion who received successful interventional recanalization treatment at Shenzhen Hospital of Southern Medical University and Heyuan People’s Hospital from January 2019 to November 2023. The Ethics Committee of Shenzhen Hospital of Southern Medical University and Heyuan People’s Hospital approved the acquisition of retrospective study patient data from the hospital’s clinical database for this study and exempted written informed consent. Inclusion criteria: (1) Baseline National Institutes of Health Stroke Scale (NIHSS) score at admission ≥6 points; (2) Preoperative Alberta Stroke Early CT (ASPECT) score ≥ 6 points; (3) The time from onset to femoral artery puncture was ≤24 h; (4) Cerebral angiography confirmed occlusion of large blood vessel in the anterior circulation; (5) The patient or family members signed and agreeded to undergo interventional recanalization treatment; (6) Interventional recanalization was successful, and the extended thrombolysis in cerebral infarction (eTICI) grading of forward blood flow was 2b-3. Exclusion criteria: (1) Previous modified Rankin scale (mRS) score ≥ 3 points; (2) Had a history of cancer, tumors or nephrotic syndrome in the past; (3) Interventional recanalization failure, eTICI grading of forward blood flow was 0-2a; (4) Cerebral angiography indicated occlusion of large blood vessel in the posterior circulation; (5) The patient or family members refused to undergo interventional recanalization treatment; (6) No blood glucose or serum albumin test was conducted at admission. The research flowchart is shown in Figure 1.

### 2.2 Treatment methods

All patients were treated according to the guidelines of the American College of Cardiology and the Stroke Association. We choosed local anesthesia or general anesthesia based on the patient’s condition and level of cooperation. After confirming the occlusion of large blood vessel in the anterior circulation during cerebral angiography, the interventional physicians selected one or more of the following methods for mechanical thrombectomy during surgery based on the patient’s lesion location, the most likely pathogenesis and the vascular pathway condition: stent thrombectomy, thrombus aspiration, balloon dilation, stent implantation and arterial use of tirofiban. The thrombectomy stents we used included Solitaire AB, RECO and TREVO. The suction catheters we used included Sofia, ACE Penumbra, and Tianxun distal access catheter. The balloon we used was Gateway balloon dilation catheter. The carotid stent we used was Protege stent. The intracranial stent we used was Apollo intracranial stent. Patients were managed according to existing guidelines after surgery.

### 2.3 Patient data collection

Trained researchers extracted demographic data and other relevant clinical data information of enrolled patients from hospital electronic databases. Follow up data was collected by trained physicians through telephone tracking or outpatient follow-up.

### 2.4 Definition and classification of variables in data

The main endpoint of this study was the incidence of futile recanalization 3 months after surgery. The secondary study endpoints were postoperative mRS score at 3 months, symptomatic cerebral hemorrhage and death within 3 months after surgery. The site of vascular occlusion can be divided into 5 categories: M1 segment of the middle cerebral artery, M2 segment of the middle cerebral artery, internal carotid artery, anterior cerebral artery and anterior circulation tandem lesion. The American Society of Interventional and Therapeutic Neuroradiology/Society of Interventional Radiology (ASITN/SIR) scoring system using Digital subtraction angiography (DSA) imaging was used to evaluate collateral circulation, with a score of 0–2 indicating poor collateral circulation and 3–4 indicating good collateral circulation. The degree of vascular recanalization was evaluated using the eTICI vascular recanalization grade. The eTICI grade of 2b-3 was defined as successful recanalization, while the eTICI grade of 0-2a was defined as failed recanalization. The pathogenesis can be divided into three types: atherosclerosis, cardiogenic embolism and other causes.

### 2.5 Calculation and grouping of AAR

The AAR calculation formula was [admission blood glucose (mmol/L) X 18 / serum albumin (g/L)] or [admission blood glucose (mg/dl) / serum albumin (g/L)], using the same blood sample obtained at admission. Patients were divided into three groups according to AAR tertile: T1 (*n* = 150; AAR ≤ 3.18), T2 (*n* = 155; 3.19 ≤ AAR ≤ 4.10), and T3 (*n* = 147; AAR ≥ 4.11).

### 2.6 Statistical processing

This study used SPSS 26.0 statistical software to conduct statistical analysis on the data. If the quantitative data conformed to a normal distribution, it was expressed as mean ± standard deviation (x ± s), and independent sample t-test was used for inter group comparison. Data that did not follow a normal distribution was described with median and interquartile intervals, and non parametric test was used for inter group comparison. Count data was expressed in percentage, and intergroup comparisons were conducted using chi square or Fisher’s exact test. For the inter group comparison of the three groups in the AAR three tertiles, the statistical analysis method of analysis of variance was used. A *p*-value less than 0.05 was considered statistically significant. Multiple logistic regression analysis were performed upon the indicators related to futile recanalization at 3 months after surgery. The statistical indicators related to futile recanalization at 3 months after surgery were included into a multiple logistic regression analysis, and stepwise backward regression analysis method was used to obtain independent factors related to futile recanalization. Receiver Operating Characteristic (ROC) curves were drawn for predicting futile recanalization at 3 months after interventional recanalization using some of independent related factors and combination of multiple independent related factors, respectively.

## 3 Results

### 3.1 Baseline clinical data of patients classified according to the AAR tertile

**Figure 2:**
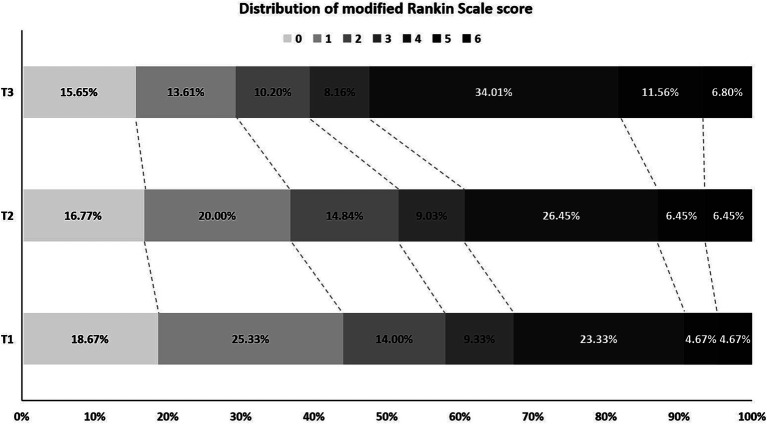
Distribution of mRS score at 3 months after successful interventional recanalization in three groups of patients according to AAR tertile. mRS, modified Rankin Scale; AAR, Admission blood glucose to serum albumin ratio.

A total of 452 patients were included and divided into three groups based on the AAR tertile. The average AAR of all patients was 4.20 ± 2.07, and the average age was 66.40 ± 12.16 years old. The baseline clinical data of the three groups of patients are shown in Table 1. Compared with T1 and T2 groups, T3 group had a higher proportion of hypertension and diabetes in the past, higher admission blood glucose, lower admission serum albumin, lower proportion of middle cerebral artery M1 segment and internal carotid artery at the occluded vessel position, higher mRS score and higher proportion of futile recanalization 3 months after surgery. As the AAR value increased, the incidence of futile recanalization tended to increase. The distribution of mRS score for 3 groups of patients at 3 months after surgery is shown in Figure 2.

**Table 1:** Clinical baseline data of T1-T3 patients categorized by AAR.

Variables	All patients (*n* = 452)	T1 (*n* = 150)	T2 (*n* = 155)	T3 (*n* = 147)	*p* value
General situation					
Age, years, (mean ± standard deviation)	66.40 ± 12.16	64.68 ± 12.76	67.06 ± 12.25	67.46 ± 11.32	0.108
Gender, Male, *n* (%)	310 (68.58)	105 (70.00)	106 (68.39)	99 (67.35)	0.884
Medical history					
Hypertension, *n* (%)	248 (54.87)	70 (46.67)	85 (54.84)	93 (63.27)	**0.016**
Diabetes, *n* (%)	85 (18.81)	10 (6.67)	14 (9.03)	61 (41.50)	**<0.001**
Cerebral infarction, *n* (%)	77 (17.04)	25 (16.67)	20 (12.90)	32 (21.77)	0.121
Coronary heart disease, *n* (%)	56 (12.39)	12 (8.00)	20 (12.90)	24 (16.33)	0.091
Atrial fibrillation, *n* (%)	81 (17.92)	24 (16.00)	34 (21.94)	23 (15.65)	0.274
Smoking, *n* (%)	177 (39.16)	58 (38.67)	59 (38.06)	60 (40.82)	0.877
Drinking, *n* (%)	101 (22.35)	30 (20.00)	35 (22.58)	36 (24.49)	0.647
Pre onset mRS ≥1, *n* (%)	34(7.52)	15(10.00)	12(7.74)	7(4.76)	0.242
The situation at the onset of stroke					
Grade of NIHSS score at admission, *n* (%)					0.969
Moderate, 6 ≤ NIHSS≤15	349 (77.21)	115 (76.67)	120 (77.42)	114 (77.55)	
Moderate to severe, 16 ≤ NIHSS≤20	82 (18.14)	28 (18.67)	29 (18.71)	25 (17.01)	
Severe, NIHSS≥21	21 (4.65)	7 (4.67)	6 (3.87)	8 (5.44)	
Preoperative ASPECT score ≤ 7, *n* (%)	227 (50.22)	74 (49.33)	73 (47.10)	80 (54.42)	0.384
Laboratory test results					
Blood glucose at admission, mmol/L, median (IQR)	7.22 (6.15–8.95)	5.87 (5.39–6.30)	7.22 (6.78–7.80)	10.20 (8.79–13.64)	**<0.001**
Serum albumin at admission, g/dL, median (IQR)	36.00 (34.00–39.00)	38.00 (36.00–40.00)	36.00 (34.00–38.00)	35.00 (33.00–37.00)	**<0.001**
Received intravenous thrombolysis treatment, *n* (%)	164 (36.28)	57 (38.00)	64 (41.29)	43 (29.25)	0.081
**Location of occluded blood vessels, *n* (%)**					**0.021**
M1 segment of middle cerebral artery	230 (50.88)	76 (50.67)	87 (56.13)	67 (45.58)	
Middle cerebral artery M2 segment	24 (5.31)	14 (9.33)	3 (1.94)	7 (4.76)	
Internal carotid artery	61 (13.50)	18 (12.00)	18 (11.61)	25 (17.01)	
Anterior cerebral artery	3 (0.66)	0 (0)	0 (0)	3 (2.04)	
Anterior circulation tandem lesion	134 (29.65)	42 (28.00)	47 (30.32)	45 (30.61)	
Good collateral circulation, *n* (%)	242 (53.54)	88 (58.67)	77 (49.68)	77 (52.38)	0.312
eTICI grade, *n* (%)					0.635
2b	98 (21.68)	36 (24.00)	34 (21.94)	28 (19.05)	
2c	23 (5.09)	10 (6.67)	7 (4.52)	6 (4.08)	
3	331 (73.23)	104 (69.33)	114 (73.55)	113 (76.87)	
Time nodes					
Time from onset to recanalization of occluded blood vessel, min, median (IQR)	543.50 (407.75–771.00)	558.50 (393.75–740.75)	521.00 (391.00–737.00)	550.00 (445.00–841.00)	0.257
Time from puncture to recanalization of occluded blood vessel, min, median (IQR)	93 (68–127)	96 (68–127.25)	91 (64–128)	93 (74–126)	0.653
**Pathogenesis**					0.158
Atherosclerotic type	226 (50.00)	66 (44.00)	75 (48.39)	85 (57.82)	
Cardiogenic embolism	120 (26.55)	42 (28.00)	45 (29.03)	33 (22.45)	
Other causes	106 (23.45)	42 (28.00)	35 (22.58)	29 (19.73)	
Safety outcomes and prognosis					
Symptomatic cerebral hemorrhage, *n* (%)	27 (5.97)	5 (3.33)	9 (5.81)	13 (8.84)	0.134
Death within 3 months after surgery, *n* (%)	27 (5.97)	7 (4.67)	10 (6.45)	10 (6.80)	0.705
mRS score 3 months after surgery, (mean ± standard deviation)	2.57 ± 1.84	2.26 ± 1.77	2.54 ± 1.84	2.93 ± 1.87	**0.007**
Futile recanalization, *n* (%)	227 (50.22)	63 (42.00)	75 (48.39)	89 (60.54)	**0.005**

AAR, Admission blood glucose to serum albumin ratio; mRS, modified Rankin Scale; NIHSS, National Institute of Health stroke scale; ASPECT, Alberta Stroke Program Early CT Score; eTICI, extended thrombolysis in cerebral infarction. Bold values in the table indicate that *p*-values less than 0.05 with a statistical difference.

### 3.2 Comparison of clinical baseline data between futile recanalization and effective recanalization groups of patients

According to the postoperative mRS score at 3 months, all patients were divided into a futile recanalization group (mRS score 3–6 points) and an effective recanalization group (mRS score 0–2 points). There were statistically significant differences between the two groups in terms of age, history of diabetes, history of coronary heart disease, history of atrial fibrillation, pre onset mRS ≥ 1, grade of NIHSS score at the time of onset, AAR tertiles, good collateral circulation, time from puncture to recanalization of occluded vessel and pathogenesis (*p* < 0.05). There was no statistically significant difference between the two groups of patients in terms of gender, history of hypertension, history of cerebral infarction, smoking history, drinking history, preoperative ASPECT score ≤ 7, admission serum albumin, receiving intravenous thrombolysis treatment, location of occluded blood vessels, eTICI grading of recanalization blood flow and the time from onset to recanalization of occluded blood vessels (*p* > 0.05). The clinical baseline data of two groups of patients are shown in Table 2.

**Table 2:** Clinical baseline data of patients with acute cerebral infarction due to large vessel occlusion of anterior circulation received successful interventional recanalization.

Variables	Effective recanalization (*n* = 225)	Futile recanalization (*n* = 227)	*p* value
General situation			
Age, years, (mean ± standard deviation)	65 (58–74)	68 (59–77)	**0.014**
Gender, Male, *n* (%)	161 (71.56)	149 (65.64)	0.175
Medical history			
Hypertension, *n* (%)	116 (51.56)	132 (58.15)	0.159
Diabetes, *n* (%)	28 (12.44)	57 (25.11)	**0.001**
Cerebral infarction, *n* (%)	36 (16)	41 (18.06)	0.560
Coronary heart disease, *n* (%)	20 (8.89)	36 (15.86)	**0.025**
Atrial fibrillation, *n* (%)	27 (12)	54 (23.79)	**0.001**
Smoking, *n* (%)	86 (38.22)	91 (40.09)	0.684
Drinking, *n* (%)	46 (20.44)	55 (24.23)	0.334
Pre onset mRS ≥ 1, *n* (%)	24 (10.67)	10 (4.41)	**0.012**
The situation at the onset of stroke			
Grade of NIHSS score at admission, *n* (%)			**<0.001**
Moderate, 6 ≤ NIHSS≤15	200 (88.89)	149 (65.64)	
Moderate to severe, 16 ≤ NIHSS≤20	21 (9.33)	61 (26.87)	
Severe, NIHSS≥21	4 (1.78)	17 (74.89)	
Preoperative ASPECT score ≤ 7, *n* (%)	110 (48.89)	117 (51.54)	0.573
AAR tertiles, *n* (%)			**0.004**
T1, AAR ≤ 3.18	87 (38.67)	64 (28.19)	
T2, 3.19 ≤ AAR ≤ 4.10	81 (36.00)	74 (32.60)	
T3, AAR ≥ 4.11	57 (25.33)	89(39.21)	
Received intravenous thrombolysis treatment, *n* (%)	77 (34.22)	87 (38.33)	0.364
Location of occluded blood vessels, *n* (%)			0.228
M1 segment of middle cerebral artery	121 (53.78)	109 (48.02)	
Middle cerebral artery M2 segment	14 (6.22)	10 (4.41)	
Internal carotid artery	33 (14.67)	28 (12.33)	
Anterior cerebral artery	1 (0.44)	2 (0.88)	
Anterior circulation tandem lesion	56 (24.89)	78 (34.36)	
Good collateral circulation, *n* (%)	202 (89.78)	40 (17.62)	**<0.001**
eTICI grade, *n* (%)			0.055
2b	39(17.33)	59(25.99)	
2c	10(4.44)	13(5.73)	
3	176 (78.22)	155 (68.28)	
Time nodes			
Time from onset to recanalization of occluded blood vessel, min, median (IQR)	550 (383.5–789.5)	537 (418–748)	0.986
Time from puncture to recanalization of occluded blood vessel, min, median (IQR)	88 (64–115.5)	103 (74–135)	**<0.001**
Pathogenesis			**<0.001**
Atherosclerotic type	117 (52)	109 (48.02)	
Cardiogenic embolism	44 (19.56)	76 (33.48)	
Other causes	64 (28.44)	42 (18.5)	

mRS, modified Rankin Scale; NIHSS, National Institute of Health stroke scale; ASPECT, Alberta Stroke Program Early CT Score; AAR, Admission blood glucose to serum albumin ratio; eTICI, extended thrombolysis in cerebral infarction. Bold values in the table indicate that *p*-values less than 0.05 with a statistical difference.

### 3.3 Multiple logistic regression analysis of futile recanalization related factors

The indicators with statistical differences in chi square test, t-test, and non parametric test were included in the multiple logistic regression analysis. These indicators included age, diabetes, coronary heart disease, atrial fibrillation, pre onset mRS ≥ 1, grade of NIHSS score at admission, AAR tertiles, good collateral circulation, time from puncture to recanalization of occluded blood vessel, and pathogenesis. Using stepwise backward regression analysis, it was found that diabetes, grade of NIHSS score at admission, AAR tertiles, good collateral circulation score and pathogenesis were independently related to futile recanalization 3 months after surgery (*p* < 0.05). Multiple logistic regression analysis is shown in Table 3.

**Table 3:** Multiple logistic regression analysis of futile recanalization after successful interventional recanalization in acute cerebral infarction with large blood vessel occlusion of anterior circulation.

Variables	Standard error	Wald	OR	95%CI	*p* value
Diabetes	0.387	4.901	0.425	0.199–0.906	**0.027**
Grade of NIHSS score at admission, *n* (%)					
Moderate, 6 ≤ NIHSS≤15	Reference	11.652	Reference	Reference	**0.003**
Moderate to severe, 16 ≤ NIHSS≤20	0.392	10.61	0.279	0.130–0.602	**0.001**
Severe, NIHSS≥21	0.794	1.969	0.328	0.069–1.556	0.161
AAR tertiles					
T1, AAR ≤ 3.18	Reference	9.954	Reference	Reference	**0.007**
T2, 3.19 ≤ AAR ≤ 4.10	0.364	0.13	1.141	0.559–2.329	0.718
T3, AAR ≥ 4.11	0.389	6.702	0.366	0.171–0.783	**0.010**
Good collateral circulation	0.329	146.399	53.748	28.188–102.484	**<0.001**
Pathogenesis					
Atherosclerotic type	Reference	4.837	Reference	Reference	0.089
Cardiogenic embolism	0.356	0.007	1.030	0.512–2.069	0.935
Other causes	0.388	4.364	2.247	1.051–4.803	**0.037**

NIHSS, National Institute of Health stroke scale; AAR, Admission blood glucose to serum albumin ratio; OR, Odds ratio; CI, Confidence interval. Bold values in the table indicate that *p*-values less than 0.05 with a statistical difference.

### 3.4 Drawing ROC curves for predicting futile recanalization

**Figure 3:**
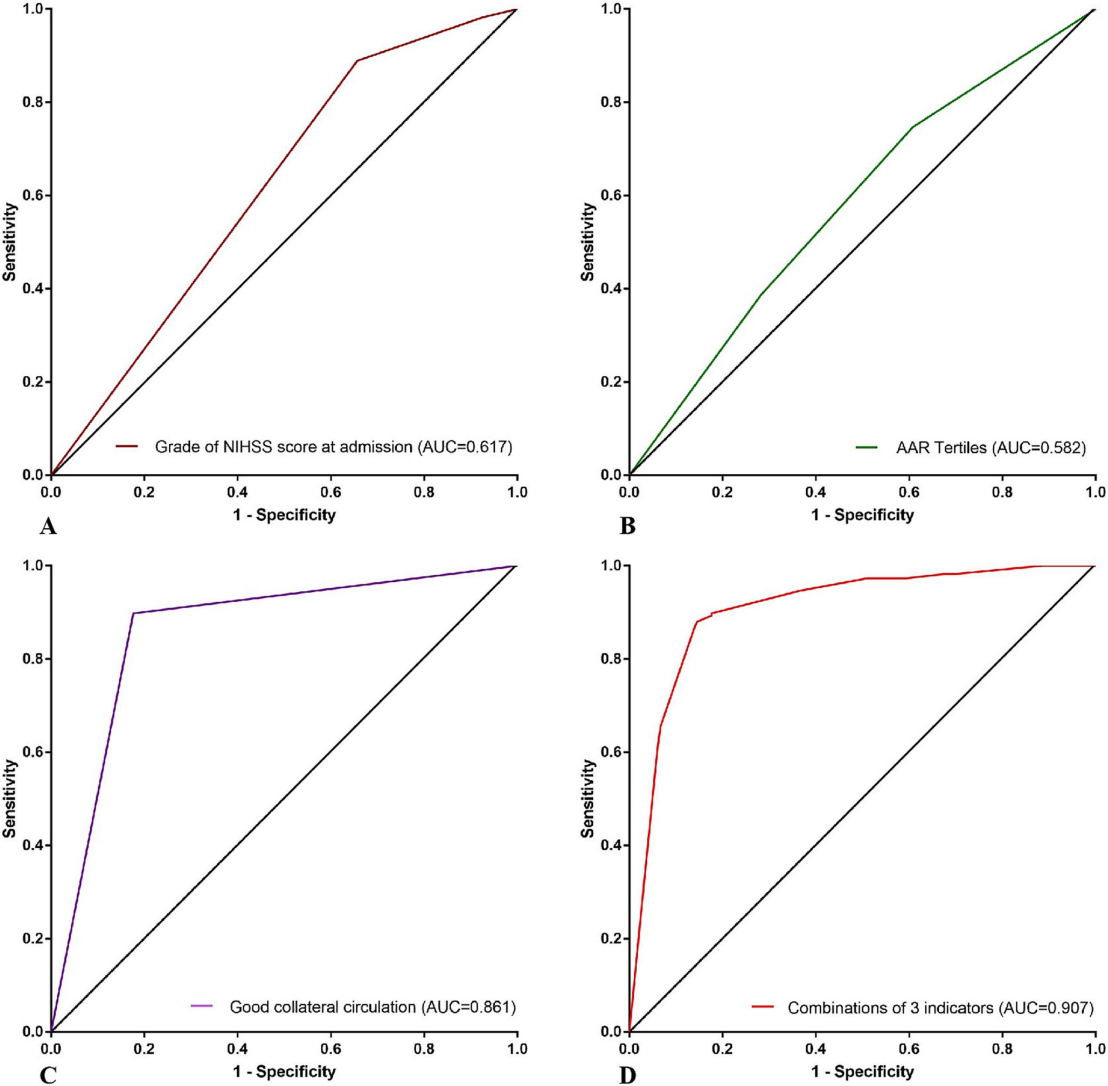
ROC curves of futile recanalization predicted by grade of NIHSS score at admission, AAR tertiles, good collateral circulation and the combination of the 3 indicators above. **(A)** ROC curve of grade of NIHSS score at admission for predicting futile recanalization after interventional recanalization. **(B)** ROC curve of AAR tertiles for predicting futile recanalization after interventional recanalization. **(C)** ROC curve of good collateral circulation for predicting futile recanalization after interventional recanalization. **(D)** ROC curve of combination of 3 indicators above for predicting futile recanalization after interventional recanalization. NIHSS, National Institute of Health stroke scale; AAR, Admission blood glucose to serum albumin ratio; AUC, Area under curve.

We plotted the ROC curves of grade of NIHSS score at admission, AAR tertiles and good collateral circulation for predicting futile recanalization after interventional recanalization. The areas under the curves (AUC) were 0.617 (95% confidence intervals (CI) 0.566, 0.669), 0.582 (95%CI 0.529, 0.634) and 0.861 (95%CI 0.824, 0.898), with sensitivity of 0.344, 0.392 and 0.824, and specificity of 0.889, 0.747 and 0.898. The ROC curve for predicting futile recanalization after interventional recanalization using a combination of grade of NIHSS score at admission, AAR tertiles and good collateral circulation was drawn. The area under the curve (AUC) was 0.907 (95% CI 0.879, 0.936) with sensitivity of 0.855 and specificity 0.880. The ROC curves of each indicator are shown in Figure 3, and the specific information of the ROC curves of each indicator is shown in Table 4.

**Table 4:** Specific information of the ROC curves of each indicator predicting futile recanalization.

Indicators	AUC	Yoden index	Truncation value	Sensitivity	Specificity	95%CI
Grade of NIHSS score at admission, *n* (%)	0.617	0.233	1.5	0.344	0.889	0.566–0.669
AAR tertiles	0.582	0.139	2.5	0.392	0.747	0.529–0.634
Good collateral circulation	0.861	0.722	0.5	0.824	0.898	0.824–0.898
Combination of 3 indicators above	0.907	0.735	0.597	0.855	0.880	0.879–0.936

ROC, Receiver Operating Characteristic curve; NIHSS, National Institute of Health stroke scale; AAR, Admission blood glucose to serum albumin ratio; AUC, Area under curve; CI, Confidence interval.

### 3.5 ROC curves for predicting futile recanalization in two groups of patients with different levels of glycated hemoglobin A1c (HbA1c)

**Figure 4:**
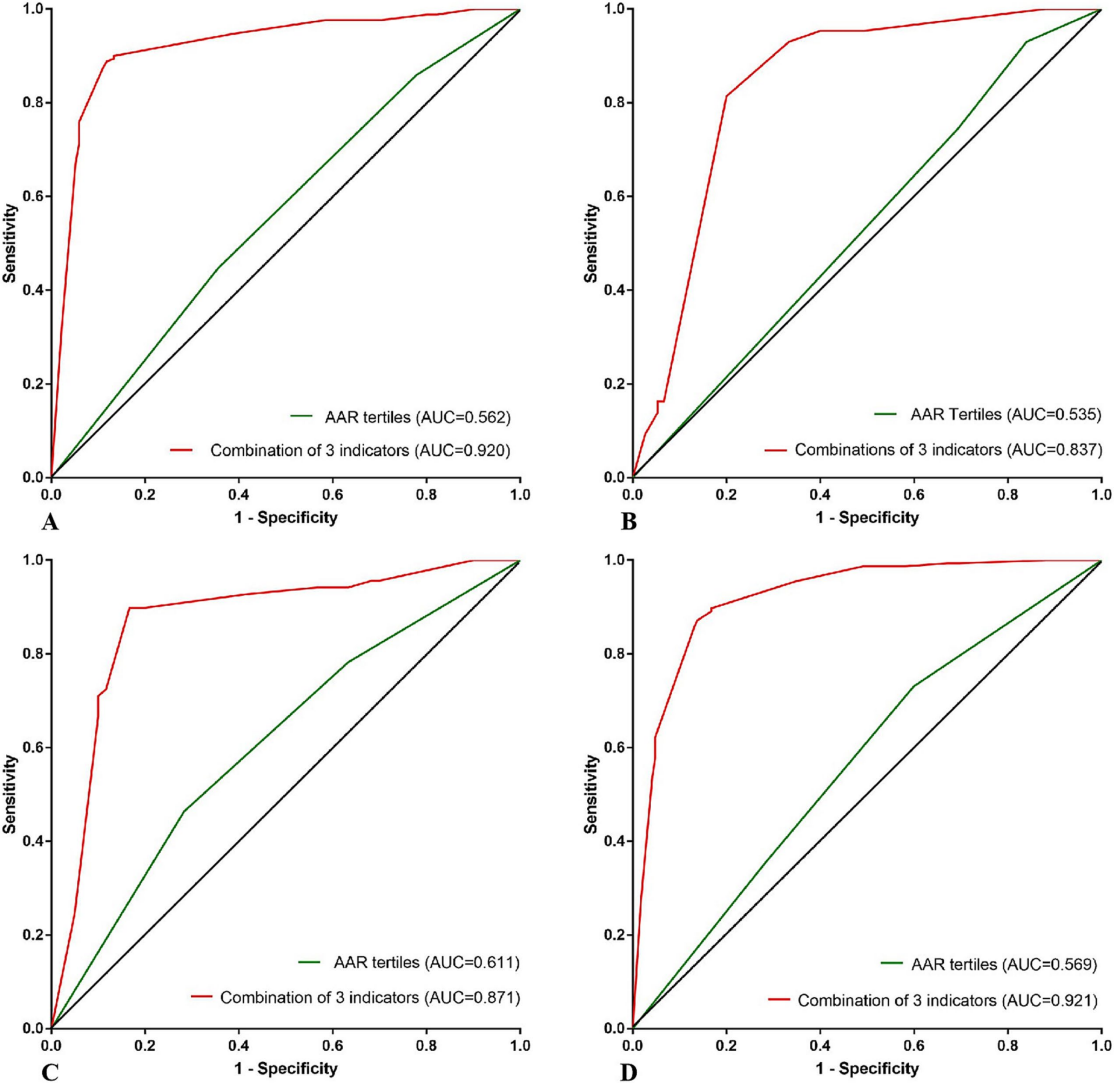
**(A–D)** ROC curves predicting futile recanalization using AAR and the combination of grade of NIHSS score at admission, AAR tertiles, good collateral circulation, respectively, according to different HbA1c of <6.5 and ≥ 6.5 IU and different ages of <60 and ≥ 60 years old. ROC, Receiver Operating Characteristic curve; AAR, Admission blood glucose to serum albumin ratio; HbA1c, glycated hemoglobin A1c; AUC, Area under curve.

According to the different levels of HbA1c, patients were divided into two groups: HbA1c <6.5 IU and HbA1c ≥ 6.5 IU. ROC curves of AAR predicting futile recanalization were plotted for both groups, with AUC values of 0.562 (95%CI 0.497, 0.627) and 0.535 (95% CI 0.429, 0.642), sensitivity values of 0.222 and 0.160, specificity values of 0.859 and 0.930. The low HbA1c group had lower sensitivity but high specificity. ROC curves predicting futile recanalization using the combination of grade of NIHSS score at admission, AAR tertiles and good collateral circulation for two groups of patients with different levels of glycated hemoglobin were drawn. The AUC was 0.920 (95% CI 0.887, 0.953) and 0.837 (95% CI 0.763, 0.912), the sensitivity was 0.881 and 0.800, the specificity was 0.888 and 0.814. The ROC curves predicting futile recanalization using the combination of grade of NIHSS score at admission, AAR tertiles and good collateral circulation in two groups of patients are shown in Figures 4A,B.

### 3.6 ROC curves for predicting futile recanalization in two groups of patients of different ages

According to different ages, patients were divided into two groups: age < 60 years old and age ≥ 60 years old. ROC curves of AAR predicting futile recanalization were plotted for both groups, with AUC values of 0.611 (95%CI 0.513, 0.708) and 0.569 (95%CI 0.506, 0.631), sensitivity values of 0.717 and 0.401, specificity values of 0.464 and 0.731. The group aged ≥60 had lower sensitivity but higher specificity. ROC curves predicting futile recanalization using the combination of grade of NIHSS score at admission, AAR tertiles and good collateral circulation for two different age groups of patients were drawn, with AUC of 0.871 (95% CI 0.804 and 0.939) and 0.921 (95% CI 0.891 and 0.952), sensitivity of 0.833 and 0.862, specificity of 0.899, 0.872. The ROC curves predicting futile recanalization using the combination of AAR, preoperative ASPECT score and collateral circulation score are shown in Figures 4C,D.

## 4 Discussion

Our study found that the ratio of admission blood glucose to serum albumin was a new composite indicator for predicting futile recanalization after successful interventional recanalization in acute cerebral infarction with anterior circulation large vessel occlusion. When patients were divided into two groups based on the level of HbA1c <6.5 IU and ≥ 6.5 IU, or into two groups based on age < 60 years old and ≥ 60 years old, AAR can still predict futile recanalization.

Emergency interventional recanalization is one of the most important and effective treatment methods for acute cerebral infarction with anterior circulation large vessel occlusion. However, major clinical studies had found that the proportion of futile recanalization after 3 months of interventional recanalization surgery for acute cerebral infarction with anterior circulation large blood vessel occlusion can reach as high as 50–60% (2–9). The existence of futile recanalization seriously affects the efficacy of interventional recanalization. It is very necessary to identify factors closely related to futile recanalization, establish a model that can be easily executed and accurately predict futile recanalization at early stage, and provide more rigorous postoperative management for high-risk patients with futile recanalization to reduce the occurrence of futile recanalization.

Blood glucose is one of the important factors affecting the prognosis of patients with acute cerebral infarction. Previous studies had shown that stress hyperglycemia after interventional recanalization was closely related to futile recanalization. By calculating the ratio of fasting blood glucose to HbA1c, it is possible to predict futile recanalization at 3 months after surgery (18–21). Besides being an indicator of nutritional status, serum albumin is also a multifunctional protein associated with inflammation and thrombosis (15–17). Previous studies had shown that serum albumin was an independent prognostic factor for ischemic stroke. The study by Wang et al. (22) suggested that higher serum albumin levels can improve the prognosis of ischemic stroke patients. Gao et al. (23) found that a decrease in serum albumin levels was independently associated with poor prognosis in patients with acute cerebral infarction of anterior circulation large blood vessel occlusion and received interventional recanalization. The experimental study by Reinhart W et al. (24) found that albumin can not only reduce the level of hematocrit of red blood cells, but also mediate red blood cell aggregation by increasing low shear viscosity and reducing red blood cell sedimentation under no flow conditions, which can help increase cerebral blood flow, rescue more neurons in the ischemic penumbra and prevent further enlargement of infarct area. At present, there is no researches using AAR to predict futile recanalization after successful interventional recanalization in acute cerebral infarction with anterior circulation large blood vessel occlusion. Admission blood glucose and serum albumin are easily obtainable indicators. Our study combined admission blood glucose levels that can reflect blood glucose stress status with serum albumin that can reflect patient nutrition, inflammation and thrombosis status, reducing potential biases when predicting using single factor. We found through statistical analysis that AAR can effectively predict futile recanalization. To our knowledge, this is the first study to apply AAR to predict futile recanalization.

The study by Yuan L et al. (25) explored the correlation between fasting blood glucose levels and clinical outcomes after endovascular treatment for ischemic stroke with anterior circulation large blood vessel occlusion in different age groups. It was found that higher fasting blood glucose levels was an independent risk factor for adverse outcomes after 3 months in patients age ≥ 60 years, but no similar effect was observed in patients age < 60 years. Our study found that patients both aged <60 years and ≥ 60 years can use AAR to predict futile recanalization. This suggested that AAR may be better at predicting futile recanalization than fasting blood glucose.

On the basis of AAR prediction of futile recanalization, we attempted to further establish a predictive model that can be easily executed and had higher sensitivity and specificity for futile recanalization. Combining the independent related indicators in multiple logistic regression analysis including grade of NIHSS score at admission, AAR tertiles and good collateral circulation, we drawn the ROC curve for predicting futile recanalization. We found that the AUC was 0.907, with sensitivity and specificity of 0.855 and 0.880, respectively, indicating high sensitivity and specificity. This prediction model can accurately identify patients with high risk of futile recanalization in the early stage. Based on the differences in glycated hemoglobin and age, we drawn ROC curves for predicting futile recanalization separately, and still found that the combined prediction of the above three indicators had a high efficiency for futile recanalization.

Because there are many factors related to futile recanalization, the statistical analysis methods we used included chi square test, t-test, non parametric test and analysis of variance. The impact of collinearity needs to be addressed in order to ultimately determine the factors independently related to futile recanalization. The multiple logistic regression analysis method we used was stepwise backward regression analysis, which can significantly reduce the impact of collinearity. However, it must be acknowledged that regardless of the use of statistical methods, there may be some errors in the final results. Therefore, it is necessary to include retrospective studies with larger sample sizes and conduct prospective clinical studies to validate the predictive efficacy of AAR in predicting futile recanalization.

This study had some limitations. Firstly, this study was a two center, retrospective study with a limited sample size. Secondly, due to the lack of clinical data for some patients, it was difficult to avoid selection bias. Thirdly, the study only included patients who received successful interventional recanalization and did not include patients who received failed interventional recanalization. Fourthly, this study only included blood glucose and serum albumin levels at admission and did not dynamically monitor blood glucose and serum albumin levels, which may not fully reflect changes in perioperative dynamic blood glucose and serum albumin levels. Fifth, the issue of multiplicity or multicollinearity in data may to some extent affect the accuracy of the results. In the future, prospective clinical studies with larger sample sizes are needed to further validate the predictive efficacy of AAR in predicting futile recanalization after successful interventional recanalization in acute cerebral infarction with anterior circulation large vessel occlusion.

## 5 Conclusion

AAR is a new composite indicator for predicting futile recanalization in patients with acute cerebral infarction with anterior circulation large vessel occlusion. The combination of grade of NIHSS score at admission, AAR tertiles and good collateral circulation has a high predictive power for futile recanalization.

## Data Availability

The original contributions presented in the study are included in the article/supplementary material, further inquiries can be directed to the corresponding authors.

## References

[1] Yaeger KA Martini ML Hardigan T Ladner T Hao Q Singh IP. Mortality reduction after thrombectomy for acute intracranial large vessel occlusion: meta-analysis of randomized trials. J Neurointerv Surg. (2020) 12:568–73. doi: 10.1136/neurintsurg-2019-015383, PMID: 31662465

[2] Goyal M Menon BK van Zwam WH Dippel DW Mitchell PJ Demchuk AM. Endovascular thrombectomy after large-vessel ischaemic stroke: a meta-analysis of individual patient data from five randomised trials. Lancet. (2016) 387:1723–31. doi: 10.1016/S0140-6736(16)00163-X, PMID: 26898852

[3] Broderick JP Berkhemer OA Palesch YY Dippel DW Foster LD Roos YB. Endovascular therapy is effective and safe for patients with severe ischemic stroke: pooled analysis of interventional Management of Stroke III and multicenter randomized clinical trial of endovascular therapy for acute ischemic stroke in the Netherlands data. Stroke. (2015) 46:3416–22. doi: 10.1161/STROKEAHA.115.011397, PMID: 26486865 PMC4659737

[4] Campbell BC Mitchell PJ Yan B Parsons MW Christensen S Churilov L. A multicenter, randomized, controlled study to investigate EXtending the time for thrombolysis in emergency neurological deficits with intra-arterial therapy (EXTEND-IA). Int J Stroke. (2014) 9:126–32. doi: 10.1111/ijs.12206, PMID: 24207098

[5] Goyal M Demchuk AM Menon BK Eesa M Rempel JL Thornton J. Randomized assessment of rapid endovascular treatment of ischemic stroke. N Engl J Med. (2015) 372:1019–30. doi: 10.1056/NEJMoa1414905, PMID: 25671798

[6] Molina CA Chamorro A Rovira À de Miquel A Serena J Roman LS. REVASCAT: a randomized trial of revascularization with SOLITAIRE FR device vs. best medical therapy in the treatment of acute stroke due to anterior circulation large vessel occlusion presenting within eight-hours of symptom onset. Int J Stroke. (2015) 10:619–26. doi: 10.1111/ijs.12157, PMID: 24206399

[7] Nogueira RG Jadhav AP Haussen DC Bonafe A Budzik RF Bhuva P. Thrombectomy 6 to 24 hours after stroke with a mismatch between deficit and infarct. N Engl J Med. (2018) 378:11–21. doi: 10.1056/NEJMoa1706442, PMID: 29129157

[8] Albers GW Marks MP Kemp S Christensen S Tsai JP Ortega-Gutierrez S. Thrombectomy for stroke at 6 to 16 hours with selection by perfusion imaging. N Engl J Med. (2018) 378:708–18. doi: 10.1056/NEJMoa1713973, PMID: 29364767 PMC6590673

[9] Olthuis SGH Pirson FAV Pinckaers FME Hinsenveld WH Nieboer D Ceulemans A. Endovascular treatment versus no endovascular treatment after 6-24 h in patients with ischaemic stroke and collateral flow on CT angiography (MR CLEAN-LATE) in the Netherlands: a multicentre, open-label, blinded-endpoint, randomised, controlled, phase 3 trial. Lancet. (2023) 401:1371–80. doi: 10.1016/S0140-6736(23)00575-5, PMID: 37003289

[10] Deng G Xiao J Yu H Chen M Shang K Qin C. Predictors of futile recanalization after endovascular treatment in acute ischemic stroke: a meta-analysis. J Neurointerv Surg. (2022) 14:881–5. doi: 10.1136/neurintsurg-2021-017963, PMID: 34544824

[11] Yang X Sun D Huo X Raynald R Jia B Tong X. Futile reperfusion of endovascular treatment for acute anterior circulation large vessel occlusion in the ANGEL-ACT registry. J Neurointerv Surg. (2023) 15:e363–8. doi: 10.1136/jnis-2022-019874, PMID: 36693725

[12] Su M Zhou Y Chen Z Pu M Li Z du H. Cystatin C predicts futile recanalization in patients with acute ischemic stroke after endovascular treatment. J Neurol. (2022) 269:966–72. doi: 10.1007/s00415-021-10680-w, PMID: 34226965

[13] Abubakar S Sabir A Ndakotsu M Imam M Tasiu M. Low admission serum albumin as prognostic determinant of 30-day case fatality and adverse functional outcome following acute ischemic stroke. Pan Afr Med J. (2013) 2:53. doi: 10.11604/pamj.2013.14.53.1941, PMID: 23565300 PMC3617615

[14] Ibrahim G Bassiouny AA El Nady H. Serum-albumin level and albumin/globulin ratio as predictors of short-term outcome of first ever ischemic stroke. Neurology. (2016) 86:238. doi: 10.1212/wnl.86.16_supplement.p5.238, PMID: 39620055

[15] Don BR Kaysen G. Serum albumin: relationship to inflammation and nutrition. Semin Dial. (2004) 17:432–7. doi: 10.1111/j.0894-0959.2004.17603.x, PMID: 15660573

[16] Bhat S Jagadeeshaprasad MG Venkatasubramani V Kulkarni MJ. Abundance matters: role of albumin in diabetes, a proteomics perspective. Expert Rev Proteomics. (2017) 14:677–89. doi: 10.1080/14789450.2017.1352473, PMID: 28689445

[17] Zhen C Chen W Chen W Fan H Lin Z Zeng L. Association between admission-blood-glucose-to-albumin ratio and clinical outcomes in patients with ST-elevation myocardial infarction undergoing percutaneous coronary intervention. Front Cardiovasc Med. (2023) 10:1132685. doi: 10.3389/fcvm.2023.1132685, PMID: 37745131 PMC10513433

[18] Zhang J Dong D Zeng Y Yang B Li F Chen X. The association between stress hyperglycemia and unfavorable outcomes in patients with anterior circulation stroke after mechanical thrombectomy. Front Aging Neurosci. (2023) 14:1071377. doi: 10.3389/fnagi.2022.1071377, PMID: 36688168 PMC9849891

[19] Sun Y Guo Y Ji Y Wu K Wang H Yuan L. New stress-induced hyperglycaemia markers predict prognosis in patients after mechanical thrombectomy. BMC Neurol. (2023) 23:132. doi: 10.1186/s12883-023-03175-w, PMID: 36997874 PMC10061963

[20] Peng Z Song J Li L Guo C Yang J Kong W. Association between stress hyperglycemia and outcomes in patients with acute ischemic stroke due to large vessel occlusion. CNS Neurosci Ther. (2023) 29:2162–70. doi: 10.1111/cns.14163, PMID: 36914967 PMC10352867

[21] Yang B Chen X Li F Zhang J Dong D Ou H. Stress hyperglycemia increases short-term mortality in acute ischemic stroke patients after mechanical thrombectomy. Diabetol Metab Syndr. (2024) 16:32. doi: 10.1186/s13098-024-01272-5, PMID: 38297321 PMC10829332

[22] Wang C Deng L Qiu S Bian H Wang L Li Y. Serum albumin is negatively associated with hemorrhagic transformation in acute ischemic stroke patients. Cerebrovasc Dis. (2019) 47:88–94. doi: 10.1159/000498855, PMID: 30897566

[23] Gao J Zhao Y du M Guo H Wan T Wu M. Serum albumin levels and clinical outcomes among ischemic stroke patients treated with endovascular Thrombectomy. Neuropsychiatr Dis Treat. (2021) 17:401–11. doi: 10.2147/NDT.S293771, PMID: 33603378 PMC7882440

[24] Reinhart W Nagy C. Albumin affects erythrocyte aggregation and sedimentation. Eur J Clin Investig. (1995) 25:523–8. doi: 10.1111/j.1365-2362.1995.tb01739.x, PMID: 7556371

[25] Yuan L Sun Y Huang X Xu X Xu J Xu Y. Fasting blood-glucose level and clinical outcome in anterior circulation ischemic stroke of different age groups after endovascular treatment. Neuropsychiatr Dis Treat. (2022) 18:575–83. doi: 10.2147/NDT.S351725, PMID: 35330823 PMC8939906

